# Synthesis of Green-Emitting (La,Gd)OBr:Tb^3+^ Phosphors

**DOI:** 10.3390/ma3042506

**Published:** 2010-04-01

**Authors:** Sun Woog Kim, Kazuya Jyoko, Toshiyuki Masui, Nobuhito Imanaka

**Affiliations:** Department of Applied Chemistry, Faculty of Engineering, Osaka University, 2-1 Yamadaoka, Suita, Osaka 565-0871, Japan; E-Mails: swkim@chem.eng.osaka-u.ac.jp (S.W.K.); kazuya@chem.eng.osaka-u.ac.jp (K.J.); masui@chem.eng.osaka-u.ac.jp (T.M.)

**Keywords:** green-emitting phosphor, rare earth oxybromide, crystal field modification, solid state reaction

## Abstract

Green-emitting phosphors based on lanthanum-gadolinium oxybromide were synthesized in a single phase form by the conventional solid state reaction method, and photoluminescence properties of them were characterized. The excitation peak wavelength of (La_1-*x*_Gd*_x_*)OBr:Tb^3+^ shifted to the shorter wavelength side with the increase in the crystal field around the Tb^3+^ ions by doping Gd^3+^ ions into the La^3+^ site, and, as a result, the green emission intensity was successfully enhanced. The maximum emission intensity was obtained for (La_0.95_Gd_0.05_)OBr:5%Tb^3+^, where the relative emission intensity was 45% of that of a commercial green-emitting LaPO_4_:Ce^3+^,Tb^3+^ phosphor.

## 1. Introduction

A number of materials activated by trivalent rare earth ions, which can exhibit light-emitting properties, have been widely used as phosphors. It is well known that some of the trivalent rare earth ions can show bright line spectra based on 4f-4f transitions, because the 4f-electrons are well shielded from the surroundings by the 5s and 5p orbitals. Among such rare earth ions, Tb^3+^ have been commonly recognized as a green activator due to the efficient emission originated from the ^5^D_4_-^7^F_J_ (J = 6, 5, 4, and 3) transitions [1].

The luminescent properties of phosphors usually depend on the concentration of the activator ion in the host lattice. When the amount of the activator is in excess, the emission intensity is decreased generally due to the concentration quenching, because the decrease in the mean activator-activator distance often induces the non-radiative deactivation. This phenomenon is significantly related to the crystal structure of the host lattice of the phosphor. Therefore, many investigations have been devoted to search for new materials, which can avoid the concentration quenching, even if a large amount of the activator is doped in the host [2,3].

In our previous studies, we reported red- and green-emitting phosphors based on rare earth oxycarbonate [4,5,6,7,8,9,10], rare earth oxysulfate [11], and zirconium oxide phosphate [12]. These phosphors can show good fluorescent properties due to their layer structures. In these structures, energy transfer from an excited luminescent ion to another across the anion groups (*i.e.,* CO_3_^2-^, SO_4_^2-^, and PO_4_^3^^-^ groups) is inhibited by the long distance. Accordingly, phosphors based on such layer structures should be resistant to concentration quenching.

**Figure 1:**
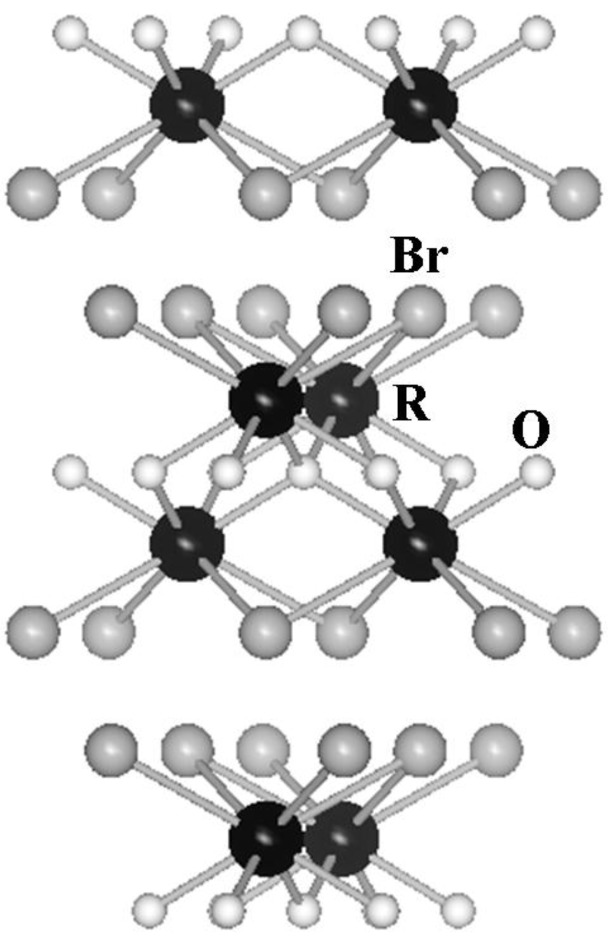
Crystal structure of the tetragonal PbFCl-type ROBr (R = rare earths).

In the present study, we have focused on tetragonal PbFCl-type rare earth oxybromide, ROBr (R = rare earths), as a host material of the phosphor. The PbFCl-type ROBr can form a layer structure similar to those of the phosphors previously studied in our laboratory, where the (RO)_n_^n+^ layers (R = rare earths) are separated by bromide ions in the direction to the *c*-axis, as illustrated in Figure 1, in which the R^3+^ ions are surrounded by four O^2-^ and four Br^-^ ions with C_4v_ point site symmetry [13,14]. In the series of the rare earth oxybromides, LaOBr has the highest thermal stability [15], and general luminescent properties of the phosphors based on LaOBr activated by trivalent rare earth ions have been investigated by several researchers [16,17,18,19,20,21,22,23,24,25,26,27,28,29,30]. Among several rare earth activators, it has been suggested that terbium is the most effective activator to obtain high emission intensity by the investigation on the X-ray luminescence of LaOBr:R^3+^ [20]. However, there is no report focusing on the relative luminescent intensities in comparison with the commercially available phosphors under UV excitation.

In this study, LaOBr:Tb^3+^ phosphors were synthesized and the photoluminescence properties of the prepared particles were investigated in detail and compared with those of the commercially available LaPO_4_:Ce^3+^,Tb^3+^ phosphor. Furthermore, in order to further enhance the emission intensity of the LaOBr:Tb^3+^ phosphor, part of the La^3+^ ions in the LaOBr host lattice were substituted with smaller Gd^3+^ ions to enhance the crystal field around the Tb^3+^ ions.

## 2. Results and Discussion

**Figure 2:**
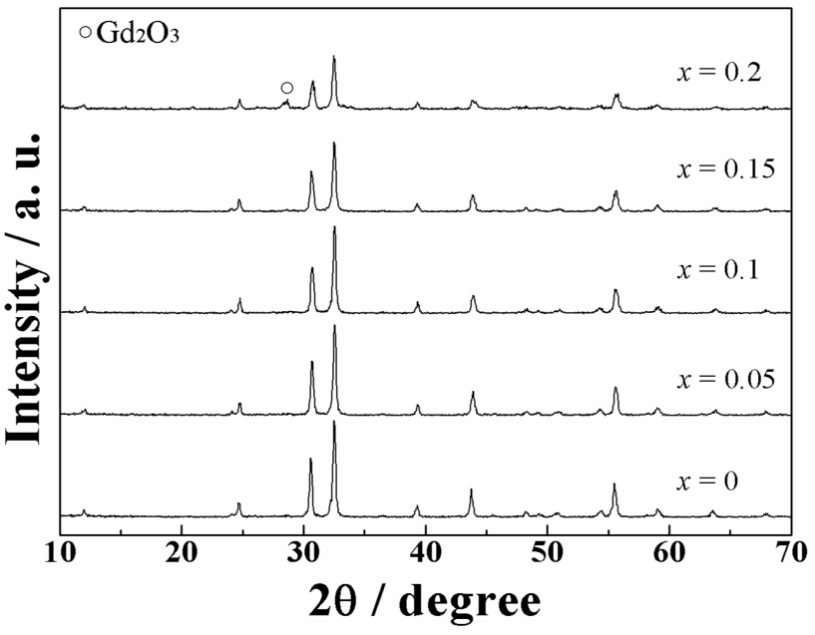
XRD patterns for the (La_1-*x*_Gd*_x_*)OBr:5%Tb^3+^ (0 ≤ *x* ≤ 0.2) phosphors.

**Figure 3:**
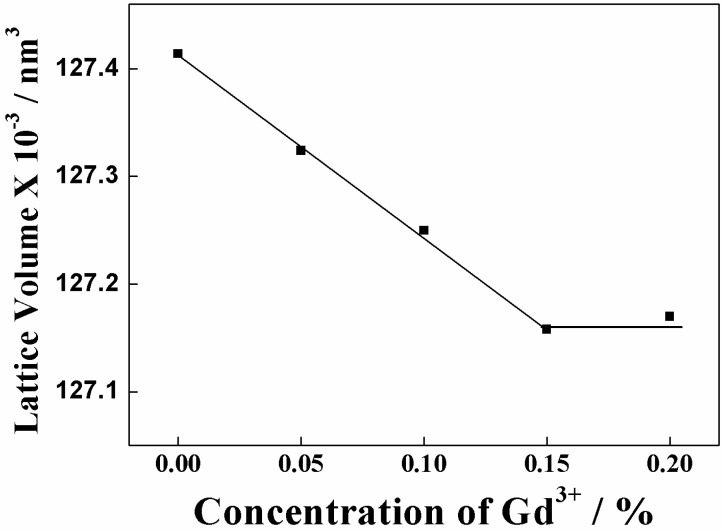
Dependence of the lattice volume on the concentration of Gd^3+^ in the (La_1-x_Gd_x_)OBr:5%Tb^3+^ (0 ≤ x ≤ 0.2) phosphors.

The sample composition was analyzed by X-ray fluorescence analysis and it was confirmed that all samples were synthesized in each stoichiometric ratio as summarized in Table 1. X-ray powder diffraction (XRD) patterns for the (La_1-*x*_Gd*_x_*)OBr:5%Tb^3+^ (0 ≤ *x* ≤ 0.2) phosphor samples are shown in Figure 2. The XRD patterns of the samples with *x* ≤ 0.15 were identical to a single phase of tetragonal PbFCl-type rare earth oxybromide structure with high crystallinity, and there is no diffraction peak corresponding to any impurities in the patterns. On the other hand, an impurity phase corresponding to cubic Gd_2_O_3_ was observed in the sample with *x* = 0.2. A peak shift to higher diffraction angle is observed with the increase in the amount of Gd^3+^ in the host LaOBr lattice for the samples with *x* ≤ 0.15, because La^3+^ (ionic radius: 0.116 nm for 8 coordination) [31] sites in the host material is partially substituted with the smaller Gd^3+^ cation (ionic radius: 0.1053 nm for 8 coordination) [31]. Figure 3 depicts the effect of Gd^3+^ content on the (La_1-*x*_Gd*_x_*)OBr:5%Tb^3+^ lattice volume, which decreases monotonously with the increase in the Gd^3+^ content and becomes approximately constant at the Gd^3+^ concentration higher than *x* = 0.15. These results indicate that the smaller Gd^3+^ successfully substituted La^3+^ site in host LaOBr lattice in the samples with *x* ≤ 0.15, and the solid solution limit is at around 15 atom % Gd^3+^ replacement of the La^3+^ site in (La_1-*x*_Gd*_x_*)OBr:5%Tb^3+^.

**Table 1:** Theoretical and analyzed compositions of the samples.

Theoretical composition	Analyzed composition
LaOBr:5%Tb^3+^	(La_0.95_Tb_0.05_)OBr
(La_0.95_Gd_0.05_)OBr:5%Tb^3+^	(La_0.9_Gd_0.05_Tb_0.05_)OBr
(La_0.9_Gd_0.1_)OBr:5%Tb^3+^	(La_0.84_Gd_0.11_Tb_0.05_)OBr
(La_0.85_Gd_0.15_)OBr:5%Tb^3+^	(La_0.79_Gd_0.16_Tb_0.05_)OBr
(La_0.8_Gd_0.2_)OBr:5%Tb^3+^	(La_0.76_Gd_0.19_Tb_0.05_)OBr

**Figure 4:**
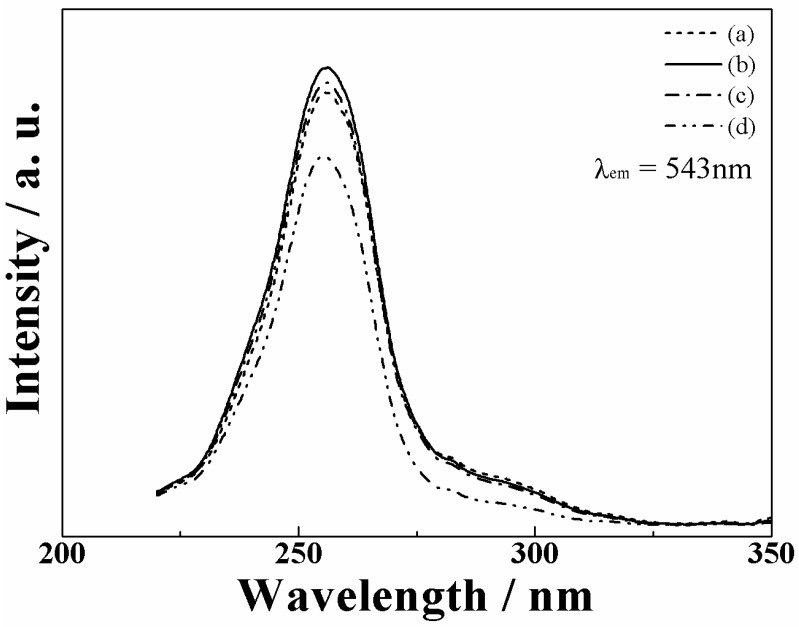
Excitation spectra of the (La_1-*x*_Gd*_x_*)OBr:5%Tb^3+^ phosphors; *x* = (a) 0, (b) 0.05, (c) 0.10, and (d) 0.15.

Figure 4 depicts the excitation spectra for the emission at 543 nm in the (La_1-*x*_Gd*_x_*)OBr:5%Tb^3+^ (0 ≤ *x* ≤ 0.15) samples. The excitation spectra of all samples consist of strong broad bands from 230 to 275 nm, corresponding to the energy transition from the 4f^8^ to 4f^7^5d configuration of Tb^3+^. The peak wavelength in the excitation bands based on the 4f-5d transition of Tb^3+^ depends on the crystal field, which is affected by the lattice volume of the oxybromide phosphor. As summarized in Table 2, each 4f-5d transition band can be divided into three Gaussian peaks, where two peaks at the short-wavelength side correspond to the spin allowed 4f-5d transitions of Tb^3+^ and the other weak peak at the long-wavelength side corresponds to the spin forbidden transition of Tb^3+^ [3234]. The crystal field strength increases with increasing amount of Gd^3+^ substitution for La^3+^ in the oxybromide lattice, because the average Tb^3+^-O^2-^ bond length becomes progressively shorter by the lattice shrinkage (Figure 3). The increase of the crystal field strength of O^2-^ around Tb^3+^ leads one of the spin allowed excitation bands to shift to shorter wavelength (higher energy), and the other one to longer wavelength (lower energy). As a result, the crystal field splitting between two spin-allowed components in Table 2 enhances with increasing the Gd^3+^ content. In addition, the energy separation between the spin-allowed and spin-forbidden component is about 6000 cm^−1^, which basically agrees with the value reported previously [32,34].

**Table 2:** Spectral data of Tb^3+^ in (La_1-*x*_Gd*_x_*)OBr:5%Tb^3+^ (*x* = 0 – 0.15).

	*x* = 0	*x* = 0.05	*x* = 0.10	*x* = 0.15
4f^8^-4f^7^5d (1) (nm)	243.9	243.4	242.8	242.1
4f^8^-4f^7^5d (2) (nm)	256.8	257.0	257.3	257.8
4f^8^-4f^7^5d (3) (nm)	282.9	283.6	284.3	284.8
Energy separation width = (1) – (3) (cm^-1^)	5652	5824	6012	6193

**Figure 5:**
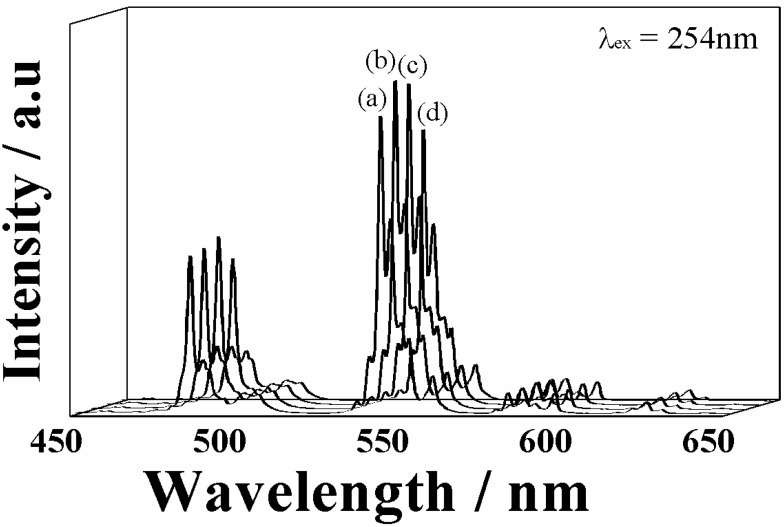
Emission spectra of the (La_1-x_Gd_x_)OBr:5%Tb^3+^ phosphors; x = (a) 0, (b) 0.05, (c) 0.10, and (d) 0.15.

Figure 5 illustrates the emission spectra of the (La_1-*x*_Gd*_x_*)OBr:5%Tb^3+^ phosphors under excitation at 254 nm. The oxybromide phosphors exhibited a well-known characteristic Tb^3+^ emission and no self-activated emission was observed in the undoped samples. The emission peaks observed at 484, 543, 587 and 625 nm correspond to the transition from the ^5^D_4_ excited level to the ^7^F_6,_^7^F_5_, ^7^F_4_, and ^7^F_3_ ground levels of Tb^3+^, respectively. In addition, the peak shape of all samples were identical with no spectral shift due to the introduction of Gd^3+^ into the LaOBr:5%Tb^3+^ lattice, because of the shielding effect of electrons in the 4f orbital by the outer 5s and 5p orbitals, whereby the crystal field has less influence.

**Figure 6:**
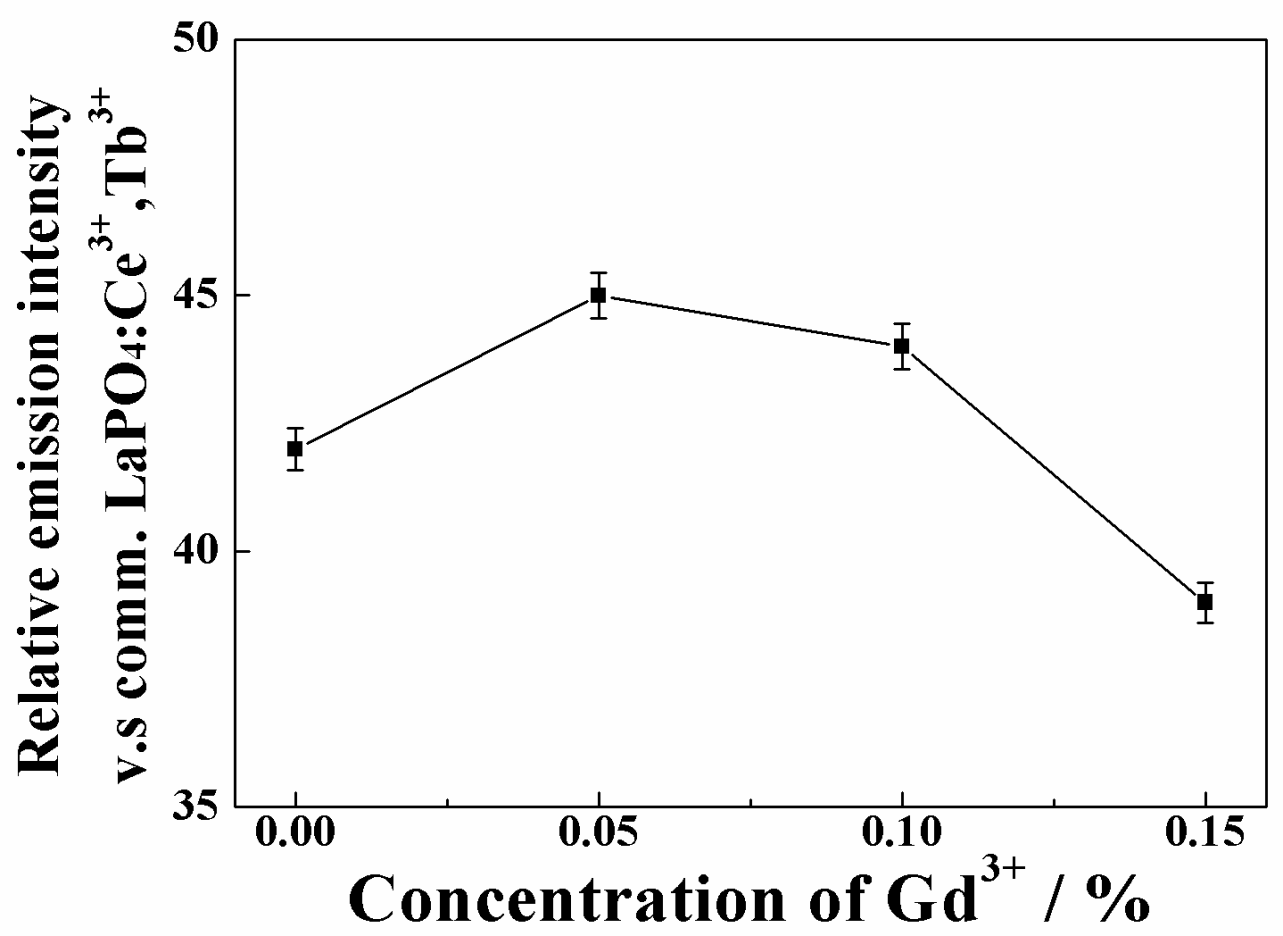
Dependence of the emission intensity on the Gd^3+^ concentration in the (La_1-*x*_Gd*_x_*)OBr:5%Tb^3+^ (0 ≤ *x* ≤ 0.15) phosphors. The excitation wavelength is 254 nm for both (La_1-*x*_Gd*_x_*)OBr:5%Tb^3+^ and LaPO_4_:Ce^3+^,Tb^3+^.

Figure 6 presents the dependence of the emission intensity on the Gd^3+^ concentration in the (La_1-*x*_Gd*_x_*)OBr:5%Tb^3+^ (0 ≤ *x* ≤ 0.15) phosphors. Reproducible results were obtained for all samples and the standard deviation, which was evaluated by statistical processing of the reproductive experiments, was small. The emission intensity was successfully increased in the samples with *x* = 0.05 and 0.10, by the Gd^3+^ doping into the LaOBr lattice.

There are two possibilities for the reason why the emission intensity increased in the samples with *x* = 0.05 and 0.10 in (La_1-*x*_Gd*_x_*)OBr:5%Tb^3+^. First, the crystal field effect is suggested. The peak wavelength in the excitation spectra shifted to shorter wavelength side and became closer to the excitation wavelength of 254 nm by the increase in the crystal field. Secondly, the increase in the crystallinity of the phosphor also contributes to the enhancement of the emission intensity. The full width at half maximum (FWHM) of the XRD peaks in Figure 2 was estimated for each (La_1-*x*_Gd*_x_*)OBr:5%Tb^3+^ (0 ≤ *x* ≤ 0.15) phosphor, and the results are summarized in Table 3. The FWHM correlates with the crystallinity of the phosphors, which affects the excitation and emission properties. The FWHM obtained for the samples with *x* = 0.05 and 0.10 became smaller than that of LaOBr:5%Tb^3+^, indicating that the crystallinity was increased by the Gd^3+^ doping. On the contrary, the FWHM for *x* = 0.15 became larger than that of LaOBr:5%Tb^3+^. These results are consistent with the excitation and emission data shown in Figure 4, Figure 5, and Figure 6. In accordance with the FWHM width of the sample, the excitation and the emission intensities reach a maximum at *x* = 0.05 in (La_1-*x*_Gd*_x_*)OBr:5%Tb^3+^.

**Table 3:** The full width at half maximum (FWHM) of the X-ray diffraction peak from the (102) planes of the (La_1-*x*_Gd*_x_*)OBr:5%Tb^3+^ (*x* = 0 – 0.15) phosphors.

	*x* = 0	*x* = 0.05	*x* = 0.10	*x* = 0.15
FWHM (degree)	0.2531	0.2457	0.2457	0.2814

The excitation and the emission intensities of the sample with *x* = 0.10 were still higher than those of LaOBr:5%Tb^3+^. However, these intensities tend to decrease with the Gd^3+^ content beyond the optimum concentration. The decrease in both the excitation and the emission intensities is probably due to the imperfect atomic arrangement correlating with the FWHM expansion in the LaOBr host matrix, and the distortion in the host matrix leads to fluorescence quenching [35]. The ionic radius of Gd^3+^ is smaller than that of La^3+^, so that the excess Gd^3+^ doping should introduce extra strain into the LaOBr lattice; therefore, emission quenching is expected beyond an optimum Gd^3+^ concentration by the decrease in the crystallinity of the phosphor.

Consequently, the maximum emission intensity was obtained at the composition of (La_0.95_Gd_0.05_)OBr:5%Tb^3+^, where the relative emission intensity was 45% compared to that of a commercial LaPO_4_:Ce^3+^,Tb^3+^ lamp phosphor. Although the emission intensity of this phosphor is not sufficient in the present stage, improvement of the luminescence property can be expected by the optimization of the amount of Tb^3+^ ion as well as the preparation process such as flux treatment, which can eliminate surface defects of the phosphor effectively. In addition, it is necessary to improve chemical stability of the oxybromide phosphor for potential application because of low stability against water. Surface treatment with AlPO_4_/Al(OH)_3_ with the additive MgSO_4_ has been suggested as an effective way to improve the stability [36].

## 3. Experimental Section

The (La_1-*x*_Gd*_x_*)OBr:Tb^3+^ (0 ≤ *x* ≤ 0.2) phosphors were synthesized by the conventional solid state reaction method. La_2_O_3_, Gd_2_O_3_, Tb(NO_3_)_3_·6H_2_O, and NH_4_Br were mixed in a stoichiometric ratio using a mortar, in which the amount of Tb^3+^ in the phosphors was adjusted to be 5%. Then, the mixture was mechanically mixed using a planetary ball milling apparatus (Pulverisette 7, FRITSCH GmbH) for 12 h. The homogenous mixture was calcined in a flow of pure N_2_ gas at 900 °C for 12 h. The precursor obtained was heated again in a flow of 2%H_2_-98%Ar gas at 900 °C for 6 h for the reduction of Tb^4+^ to Tb^3+^. 

The crystal structure of the samples was identified by X-ray powder diffraction (XRD; Rigaku Multiflex) analysis, and the sample composition was confirmed by X-ray fluorescence spectroscopy (Rigaku ZSX100e). Photoluminescence excitation and emission spectra were measured at room temperature with spectrofluorophotometer (Shimadzu RF-5300PC). The emission spectra were obtained for excitation at 254 nm, and the excitation spectra were recorded for the emission peak at 543 nm (^5^D_4_-^7^F_5_ transition of Tb^3+^). The relative emission intensities of the (La_1-*x*_Gd*_x_*)OBr:5%Tb^3+^ (0 ≤ *x* ≤ 0.2) phosphors were estimated by comparing the integrated area of the emission peak at 543 nm with that of the commercial LaPO_4_:Ce^3+^,Tb^3+^ phosphor.

## 4. Conclusion

Green-emitting phosphors based on lanthanum-gadolinium oxybromide, (La_1-*x*_Gd*_x_*)OBr:5%Tb^3+^ (0 ≤ *x* ≤ 0.2) were synthesized by the conventional solid state reaction method. Oxybromide phosphors with tetragonal PbFCl-type structure were obtained in a single phase having high crystallinity for the samples with *x* ≤ 0.15. The photoluminescence spectra showed green emission from the ^5^D_4_ excited level to the ^7^F*_J_* (*J* = 6, 5, 4, and 3) ground levels of Tb^3+^. The photoluminescent intensity was increased by the Gd^3+^ doping and the emission reached the maximum intensity at *x* = 0.05 in (La_1-*x*_Gd*_x_*)OBr:5%Tb^3+^, where the relative emission intensity compared with that of the commercial LaPO_4_:Ce^3+^,Tb^3+^ phosphor was 45%.
